# Regulatory roles of E3 ubiquitin ligase RNF128 in inflammatory diseases and tumors

**DOI:** 10.7150/jca.138010

**Published:** 2026-09-05

**Authors:** Qiuxiang Xiao, Qidong Liu, Jing Wei, Dalang Yu

**Affiliations:** 1 Department of Pathology, The First Affiliated Hospital of Gannan Medical University, Ganzhou, Jiangxi, China.; 2 School of Basic Medicine, Fuzhou Medical College, Fuzhou, Jiangxi, China.

**Keywords:** RNF128, ubiquitination, inflammation, tumors, roles

## Abstract

Ring finger protein 128 (RNF128) belongs to the family of transmembrane E3 ubiquitin (Ub) ligases. It mediates substrate-specific ubiquitination as a critical post-translational modification and regulates diverse physiological activities and pathological processes. More importantly, the E3 Ub ligase RNF128 is involved in both innate and adaptive immune responses through ‌Ub-dependent regulation‌ of its target proteins. Dysregulation of RNF128 is associated with the occurrence and development of multiple diseases. In recent years, numerous studies have demonstrated the involvement of RNF128 in the progression of inflammatory disorders and tumors. Nevertheless, mechanistic discrepancies persist regarding its dual pro-/anti-inflammatory and pro-/anti-tumor functions. Furthermore, there remains a lack of systematic evaluation concerning the ‌context-dependent regulatory roles of RNF128 in inflammatory responses and tumorigenesis, as well as its targeted therapeutic landscape. Accordingly, this article comprehensively reviews the structure-function relationships of RNF128, its mechanistic regulation of inflammation and tumor progression, and the existing functional controversies in colorectal cancer. It summarizes potential targeted intervention strategies and clinical translational prospects, highlights current research limitations, and proposes future investigative directions. This review aims to provide scientific references for further mechanistic exploration of RNF128 and the development of precision therapeutic strategies for related diseases.

## Introduction

Ubiquitination governed by ‌the‌ ubiquitin (Ub)-proteasome system (UPS) serves as one of the most vital post-translational modifications of proteins, modulating diverse physiological and pathological events ‌in eukaryotes‌. Ub is a small 76-amino-acid protein highly conserved across eukaryotes. It contains seven functional lysine residues (K6, K11, K27, K29, K33, K48, and K63) and one N-terminal residue (Met1) 1. The covalent attachment of Ub to substrate proteins is achieved through the E1, E2, and E3 cascade. Firstly, an E1-activating enzyme forms a thioester intermediate with the C-terminal Gly of Ub, and Ub is then transferred to the active-site Cys on one of E2 Ub-conjugating enzymes, resulting in another thioester intermediate, E2-Ub 1. Finally, an E3 Ub ligase binds the E2-Ub and the substrate to facilitate the formation of an isopeptide bond between the C-terminal carboxyl of Ub and the ε-amino group of a lysine side chain or free N-terminal amino group of the substrate 2. Based on distinct linkage patterns, Ub can form monoubiquitination, multi-monoubiquitination, diverse polyubiquitin chains, and branched polyubiquitin chains within cells 3, 4. The biological outcomes of ubiquitination are largely dictated by the linkage modes of Ub chains, chain lengths, as well as additional post-translational modifications attached to Ub moieties 5. Ub chains linked via K6 are implicated in cellular DNA damage responses. K11-linked Ub chains participate in the regulation of cell-cycle progression, proteasomal degradation and intracellular membrane trafficking. K27-linked Ub chains take part in protein secretion modulation, DNA-damage repair and mitochondrial stress responses. Both K29- and K48-linked Ub chains predominantly facilitate substrate degradation through the proteasome pathway. K33-linked Ub chains are capable of restraining type I interferon (IFN) signaling and regulating intracellular protein transport. Besides their role in DNA-damage repair, K63-linked Ub chains are also utilized for selective autophagy through adaptors such as p62/SQSTM1. In addition, Met1-linked linear Ub chains activate nuclear factor-κB (NF-κB) signaling while suppressing interferon-related signal transduction 3, 6-8. Importantly, E3 Ub ligases are responsible for substrate-specific recognition, thus controlling most of the aforementioned biological activities 5. Beyond catalyzing Ub-dependent reactions, E3 Ub ligases also govern cell-cycle control, DNA-damage repair, intracellular signal transduction, and immune surveillance 9. Collectively, abnormal expression ‌and‌ dysfunction of E3 Ub ligases ‌are‌ closely associated with the onset and progression of inflammatory diseases and cancers, highlighting their critical contributions to pathological processes.

Ring finger protein 128 (RNF128), also known as gene related to anergy in lymphocytes protein (Grail), is a transmembrane E3 Ub ligase. It is broadly expressed in immune cells,‌ liver, ‌and‌ colonic tissues, as well as in other cell types and organs, and participates in the regulation of several immune responses. In CD4^+^ T cells, elevated RNF128 expression facilitates Ub-mediated degradation of the intracellular TCR-CD3 complex, thus limiting T cell activation and sustaining T cell immune tolerance 10. RNF128 protects macrophages against severe lipopolysaccharide-induced inflammatory injury in mouse models 11. RNF128 overexpression has been shown to promote the proliferation, migration, invasion, and apoptosis resistance of hepatocellular carcinoma (HCC) cells both *in vitro* and *in vivo* 12. In addition, RNF128 overexpression suppresses colorectal cancer (CRC) cell proliferation, migration, and invasion, while its knockout exerts opposing effects 13. In recent years, increasing evidence has suggested that RNF128 plays an important regulatory role in the development of inflammatory diseases and tumorigenesis. Therefore, deciphering precisely how RNF128 drives these pathologies will be critical for manipulating RNF128 activity, which may allow accurate and efficient modulation of immune-driven pathological processes. More importantly, such insights may facilitate the development of novel, ‌precisely targeted therapeutic approaches for related clinical diseases.

## 1. Structure and function of the E3 Ub ligase RNF128

### 1.1 Structural and ubiquitination-derived degradation processes of the E3 Ub ligase RNF128

**Figure 1:**
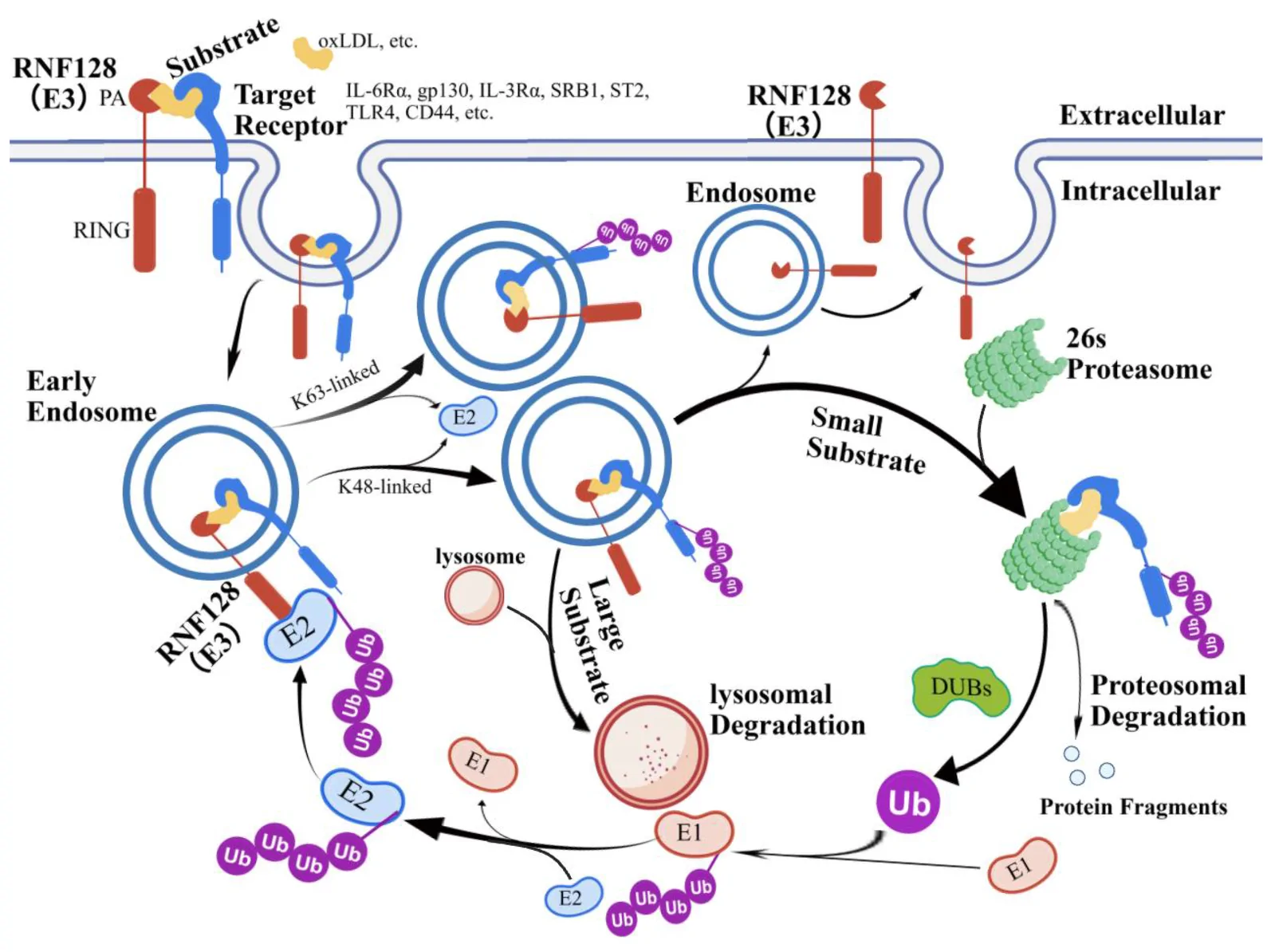
** RNF128 and the protein degradation network.** RNF128 selectively mediates the ‌internalization‌ of extracellular substrates and target receptors, ‌followed by their ubiquitination‌. The K63 polyubiquitin chain represents a non-degradative ubiquitination, whereas the K48 polyubiquitin chain represents a degradative ubiquitination. During protein degradation, small soluble substrates and target receptors are degraded via the 26S proteasome, whereas‌ Ub conjugated to these substrates undergoes‌ deubiquitination and re-enters the Ub cycle. RNF128 localized in the endosomes is then transported back to the cell membrane. In contrast, large substrates and target receptors modified by ubiquitination are degraded through the lysosomal pathway.

Based on structure and functional differences, E3 Ub ligases are broadly classified into three major groups: the Really Interesting New Gene (RING) finger type, the Homologous to the C-terminus of E6-associated protein (HECT) type, and the U-box type 14. The RING finger type is the most predominant class, distinguished by a highly conserved RING domain, and can be further subdivided into five subfamilies: Membrane-Associated RING-Cysteine-Histidine (MARCH), Protease-Associated (PA)-Transmembrane (TM)-RING, RING-between-RING (RBR), Ub-Interacting Motif (UIM), and Tripartite Motif (TRIM) 15. RNF128 belongs to the PA-TM-RING subfamily and is known as a receptor eliminated through E3 Ub ligase recruitment (REULR) 16. It is composed of 428 amino acids, localized in the endosomal recycling pathway, and includes three main structural domains: an extracellular (N-terminal) PA domain, a TM domain, and an intracellular (C-terminal) RING domain 9, 16. The RING domain consists of Cys and His residues that coordinate two Zn^2+^ ions, mostly in a cross-brace arrangement. It provides a docking site for E2 Ub-conjugating enzymes, enhancing Ub transfer to substrates 17. Interestingly, the RING domain of RING-type E3 Ub ligase does not possess a catalytic Cys at the active site. Thus, it does not form high-energy thioester intermediate, unlike HECT and RBR E3 Ub ligases 17. This indicates that RNF128 primarily acts as a scaffold protein, facilitating the transfer of Ub. These domains together mediate essential biological processes, including protein ubiquitination and the regulation of protein degradation. In particular, RNF128 utilizes its PA domain to mediate the entry of target proteins (including cell surface receptors) into the endocytic pathway, a step that is a prerequisite for subsequent ubiquitination 15. During ubiquitination, the RING domain of RNF128 facilitates the binding of ‌an‌ E2 Ub-conjugating enzyme, enabling the direct transfer of Ub from the E2 to substrates without forming an E3-Ub intermediate. ‌Subsequently‌, the ubiquitinated substrates are degraded by ‌the proteasome‌ pathway 9. After substrates bound to the proteasome, deubiquitinating enzymes (DUBs) catalyze deubiquitination, and the ‌released Ub re-enters the Ub cycle‌ to maintain a steady supply of free Ub for future reactions 18. Generally, small soluble substrate proteins are degraded by the 26S proteasome. In contrast, large substrate proteins, after undergoing ubiquitination, are sorted into endosomes, transported, and ‌delivered to lysosomes via membrane fusion‌ for ultimate degradation and clearance 18 **(Figure 1)**.

### 1.2 Structure-function relationships of the E3 Ub ligase RNF128

RNF128 primarily relies on its PA domain to specifically bind to ‌cell surface receptors‌ or target proteins, driving endocytosis of target substrates into the endosomal system and ‌priming these substrates‌ for subsequent ubiquitination 19. The TM domain anchors RNF128 to the membrane, sustaining its stable distribution within the endosomal recycling pathway and ensuring continuous substrate recruitment and modification 20. The intracellular RING domain serves as the core catalytic region, directly binding to an E2 Ub-conjugating enzyme‌ and mediating ‌the‌ transfer of Ub‌ molecules to substrate proteins without forming an E3-Ub intermediate, thereby rapidly initiating the ubiquitination process 9. These structural features are the structural basis for substrate-specific recognition, differential ubiquitination, and bidirectional functional regulation.

RNF128 selectively mediates different types of Ub chain modifications to achieve bidirectional regulation of inflammation and tumor progression. Among diverse ‌ubiquitination forms‌, K48-linked and K63-linked chains constitute the major classical modification modes deployed by RNF128, each with highly specialized functions. K48-linked Ub chains mark substrates for canonical proteolytic degradation. RNF128 targets substrates including interleukin-6 receptor α (IL-6Rα) and glycoprotein 130 (gp130), ‌driving their‌ K48-linked ubiquitination ‌and subsequent‌ degradation via the 26S proteasome or lysosomal routes. ‌This event dampens‌ the pro-inflammatory and pro-tumor IL-6-STAT3 pathway, ‌thereby restraining‌ inflammation and tumorigenesis 21. In contrast, ‌K63-linked Ub chains confer‌ non-proteolytic ubiquitination that mainly govern ‌signaling‌-pathway activation and protein-protein interaction dynamics‌. RNF128 catalyzes ‌K63-linked ubiquitination‌ of TBK1, which promotes‌ TBK1-IRF3 pathway activation, triggers IFN-β secretion, and positively modulates host innate immune responses 22. Selective switching between these two classical Ub-chain types constitutes the key mechanism enabling RNF128 to balance its tumor-suppressive and immune-activating functions.

Beyond classical ubiquitination, RNF128 also mediates non-classical ubiquitination to fine-tune intracellular signaling. K27-linked Ub chains constitute a critical non-classical ubiquitination governing innate immunity and inflammatory responses. By driving K27-linked ubiquitination of target proteins, RNF128 modulates membrane-receptor-dependent inflammatory signaling and fine-tunes chronic inflammatory microenvironment 23. Regrettably, there are currently no reported studies investigating the role of RNF128 in the ubiquitination processes involving K11, K29, K33, K6, and Met1. These constitute key unresolved gaps within the RNF128-centered Ub regulatory network. In recent years, research on the structure and function of RNF128 homologous proteins such as RNF167 and RNF149 in the PA-TM-RING family has gradually deepened. Structurally, members of this homologous protein class all possess the conserved PA-TM-RING modular structure. The intracellular RING domain retains‌ robust Ub‌-catalytic activity, whereas sequence and conformational divergence within the ‌PA‌ domain confer substrate specificity and functional diversification. Functionally, RNF167 catalyzes non-classical K6- and K11-linked ubiquitination ‌of‌ RIG-I and MDA5, inhibiting IFN-I overactivation via ‌both lysosomal and proteasomal pathways 24. RNF149 mediates ‌K29-linked ubiquitination and subsequent degradation‌ of CD63, thereby suppressing ‌the lipopolysaccharide/Toll-like receptor 4 (LPS/TLR4) inflammatory signaling cascade‌ 25. Based on this, it is inferred that RNF128 may also exhibit a non-classical ubiquitination similar to that of its family homologous proteins in immune regulation. Collectively, RNF128 assembles a substrate-specific regulatory network by differentially activating both classical and non-classical ubiquitination, elucidating the molecular underpinnings of its disease-dependent functional heterogeneity **(Table 1)**.

In summary, the unique structural characteristics and diverse ubiquitination patterns of RNF128 determine its context-dependent functional plasticity. The distinct substrate preferences and regulatory modes described above largely account for its dual pro-inflammatory/anti-inflammatory and pro-tumor/anti-tumor behaviors across different disease contexts. The following sections further elaborate on how these mechanistic features drive the pathological progression of multiple inflammatory disorders and tumors, and explore the corresponding targeted therapeutic applications.

## 2. Regulatory roles and mechanisms of the E3 Ub ligase RNF128 in inflammatory diseases

### 2.1 Regulation of immune tolerance in autoimmune diseases

**Figure 2:**
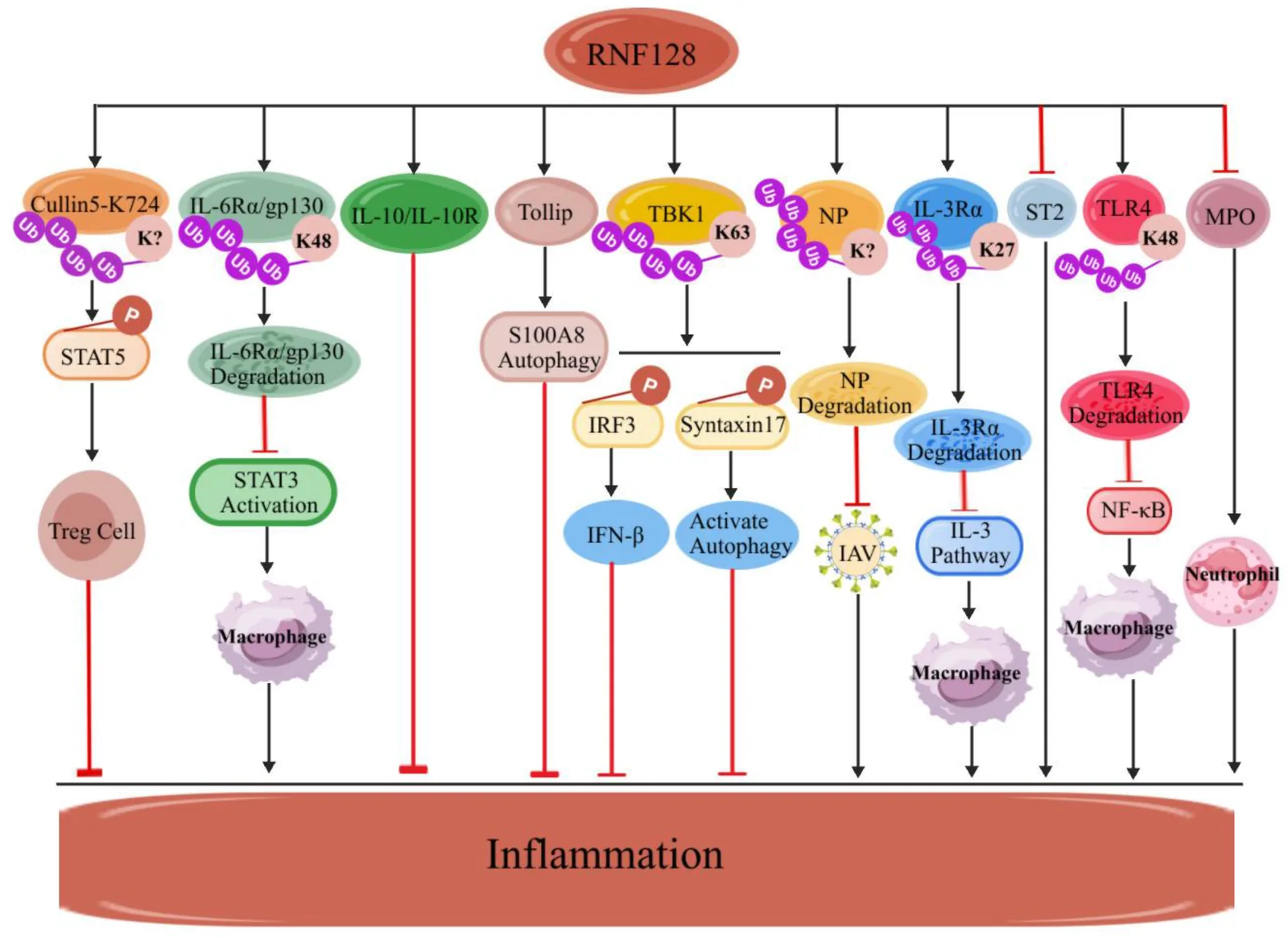
** RNF128 anti-inflammatory mechanisms.** RNF128 mediates the ubiquitination of Cullin5, thereby activating STAT5 signaling and subsequently promoting the negative regulation of inflammation by Treg cells; RNF128 facilitates the ubiquitination-mediated degradation of IL-6Rα/gp130, NP, IL-3Rα, and TLR4, thereby inhibiting signaling pathways such as those involving STAT3, IL-3, and NF-κB to exert an anti-inflammatory effect; RNF128 suppresses the inflammatory process by regulating IL-10R expression and modulating the Tollip-S100A8 axis, as well as the activation of the TBK1/IRF3/IFN-β and TBK1/Syntaxin17/autophagy signaling pathways; RNF128 counteracts inflammation by inhibiting ST2 and MPO.

The central process involved in the pathogenesis of autoimmunity is immunodysregulation defined as suboptimal T cell activation and, more importantly, defective suppressive activity of regulatory T cells (Tregs). Particularly, the Treg defect seen in autoimmune disorders is not due to low cell numbers, but instead to defects in interleukin-2 receptor (IL-2R) signaling. These signaling defects further lead to the loss of inhibitory control over IL-2R desensitization 26. Under ‌physiological conditions‌, RNF128 mediates the ubiquitination of ‌residue K724‌ in Cullin5, ‌thereby blocking‌ its deacetylation and preventing the degradation of phosphorylated JAK1 (p-JAK1) and the DEP-domain-containing mTOR-interacting protein (DEPTOR). Consequently, this maintains p-STAT5 transcriptional activity, prevents IL-2R desensitization within Tregs, and augments their suppressive function 26. Previous studies have demonstrated a significant downregulation of RNF128 expression in peripheral blood mononuclear cells and CD4⁺ T cells isolated from patients with systemic lupus erythematosus (SLE) 27. In the absence of RNF128, both p-JAK1 and DEPTOR are degraded, causing disrupted IL-2R signaling, leading to defective Treg functionality and subsequent autoimmune disease progression 28. Restoration of‌ Treg functionality could ‌re-establish‌ normal inhibition of IL-2R desensitisation through ‌the blockade of Cullin5 deformylation, thus sustaining immune tolerance. This mechanism could represent an effective therapy for ‌autoimmune diseases‌. Furthermore, RNF128 overexpression can induce T cell dysfunction via NK cells, thereby alleviating the progression of experimental autoimmune encephalomyelitis (EAE) 29. Based on these findings, it can be inferred that RNF128 may also suppress the progression of autoimmune diseases such as rheumatoid arthritis and psoriasis. Nevertheless, therapeutic research targeting RNF128 for these conditions remains limited. To summarize, RNF128 ubiquitinates ‌specific substrates within‌ immune cells to modulate T cell activity, ‌thereby‌ affecting the onset and progression of autoimmune diseases **(Figure 2)**.

### 2.2 Regulation of inflammatory bowel disease (IBD) progression

IBD is a chronic inflammatory disease caused by aberrant host immune-system attacks against intestinal tissues. It mainly includes ulcerative colitis (UC) and Crohn's disease (CD). Growing evidence supports a role for RNF128 in ‌the‌ development of IBD. In UC patients, RNF128 expression in peripheral blood CD4^+^ T cells is remarkably upregulated during ‌the‌ remission phase relative to ‌the‌ activity phase, and its expression level correlates with favorable clinical responses following treatment, indicating ‌its‌ potential involvement in UC resolution 30. On the other hand, CD patients exhibit lower RNF128 abundance in peripheral blood CD4^+^ T cells relative to controls, independent of disease activity 31. These observations indicate that RNF128 may restrain intestinal inflammation, whereas its expression patterns in peripheral blood CD4⁺ T cells differ substantially between UC and CD patients. Given the scarcity of gene-knockout-based mechanistic investigations, how RNF128 functions in IBD remains incompletely elucidated. However, recent studies have revealed that RNF128 facilitates K48-linked polyubiquitination of IL-6Rα and gp130, mediating their translocation to lysosomes for degradation. This event blunts ‌‌signal transducer and activator of transcription 3 (STAT3) activation and mitigates colonic inflammatory‌ injury 21. Another report demonstrates that RNF128 in CD4^+^ T cells sustains the pro-/anti-inflammatory balance critical for intestinal homeostasis. RNF128-deficient CD4^+^ T cells from colonic mucosal display reduced interleukin-10 (IL-10), and its receptor (IL-10R) levels, accompanied by decreased microbial diversity and enrichment of pro-inflammatory commensals 32. Together, these results indicate that RNF128 confers intestinal protective effects via regulating IL-10R to promote‌ regulatory immune responses and modulate‌‌ intestinal microbiota. Nevertheless, the precise mechanism by which RNF128 exerts its Ub-mediated degradation function within the intestine remains not fully elucidated. In macrophages, RNF128 deficiency suppresses Toll-interacting protein (Tollip) dependent cargo-receptor-mediated autophagic degradation of calcium-binding protein A8 (S100A8). Elevated S100A8 levels consequently boost inflammatory factors secretion, accelerating the onset of colonic inflammation 33. All these findings highlight the critical role of RNF128 in IBD regulation **(Figure 2)**.

### 2.3 Regulation of cardiovascular diseases (CVD) progression

**Figure 3:**
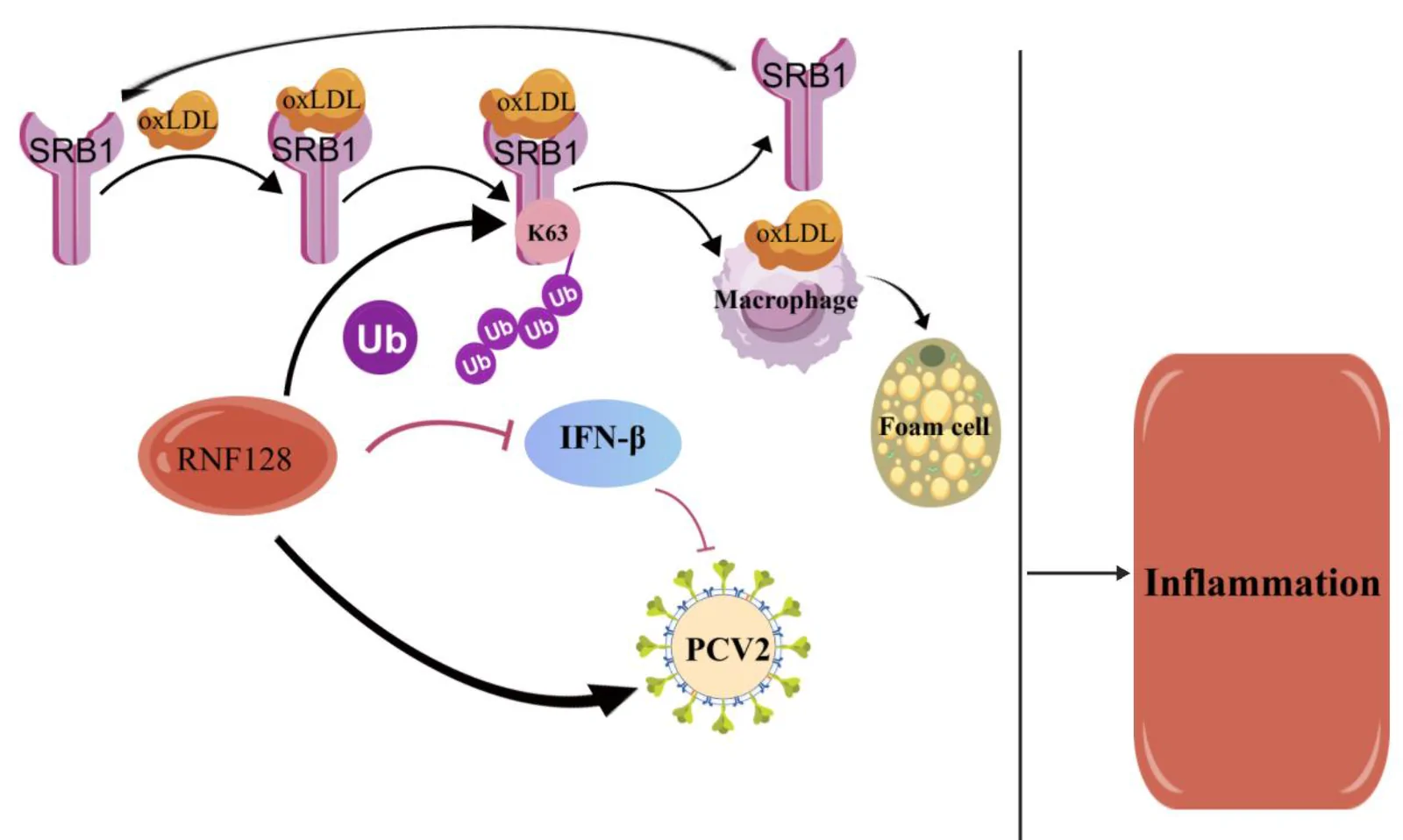
** RNF128 pro-inflammatory mechanisms.** RNF128 induces foam cell formation by ubiquitinating SRB1 to increase oxLDL uptake, thereby playing a pro-inflammatory role. Additionally, RNF128 suppresses antiviral IFN-β production, facilitating PCV2 replication and triggering inflammation.

Inflammation is an independent risk factor for atherosclerotic cardiovascular disease (ASCVD), with currently available pharmacological anti-inflammatory interventions exhibiting limited efficacy 34. Accordingly, novel anti-inflammatory strategies are urgently required to counter ASCVD-related inflammatory responses. Recent studies have shown that macrophages and T cells are the predominant cellular constituents within human atherosclerotic (AS) plaques 35, 36. The generation of macrophage-derived foam cells is a defining hallmark of AS lesions. Sustained hyperlipidemia induces high expression of RNF128 in macrophages, and macrophage-specific RNF128 deletion alleviates AS in both male and female mice 37. Mechanistic investigations have demonstrated that RNF128 knockout in macrophages decreases endocytosis of oxidized low-density lipoprotein (oxLDL), thus inhibiting foam cell formation. Mechanistically, RNF128 recognizes the extracellular domain of scavenger receptor B1 (SRB1) via its PA domain and promotes K63-linked ubiquitination of SRB1 at lysine 478. This ubiquitination facilitates SRB1 transport to the plasma membrane via Rab11, allowing it to evade lysosomal degradation. This process enhances SRB1-mediated oxLDL uptake, promoting ‌foam cell formation‌ 37. Consequently, the E3 Ub ligase RNF128 drives foam cell formation and exacerbates CVD-related inflammation **(Figure 3)**.

### 2.4 Regulation of microbial infection occurrence and development

In recent years, studies have confirmed that RNF128 plays an important role in the host immune response to microbial infection **(Figures 2 and 3)**. In terms of antiviral immunity, RNF128 is a critical positive regulatory factor. Deficiency of RNF128 impairs interferon regulatory factor 3 (IRF3) activation and IFN-β signaling 22. Furthermore, additional mechanistic studies have shown that the PA domain of RNF128 binds to TANK-binding kinase 1 (TBK1), and its RING domain catalyzes K63-linked ubiquitination of TBK1. Ubiquitination at this site does not directly induce protein degradation; instead, it promotes TBK1 activation via conformational rearrangement or adaptor-protein recruitment. Activated TBK1 phosphorylates IRF3, thus inducing the expression and secretion of the antiviral cytokine IFN-β 22. In addition, RNF128 catalyzes ubiquitination of viral nucleoprotein (NP), targeting NP for‌ proteasomal degradation and consequently restraining viral replication 38. Conversely, after *Porcine circovirus type 2* (PCV2) induces RNF128 expression, RNF128 inhibits IFN-β production and enhances PCV2 replication in porcine epithelial cells (PK15 cells) 39. Mechanistically, the upregulation of RNF128 is mediated by PCV2 open reading frame 5 (ORF5), rather than by pattern recognition receptor-mediated signal transduction in PK15 cells 39. Unfortunately, this study did not explain in detail how ORF5 induces RNF128 expression or describe the process by which RNF128 inhibits IFN-β production. Based on the properties of ‌RNF128 as an E3 Ub ligase‌, it can be inferred that upregulation of RNF128 does not occur through direct binding of ORF5 to the RNF128 gene promoter as a transcription factor, but rather through a cellular signaling cascade following viral infection or via mediation by host factors. Upregulated RNF128 could ‌catalyze the ubiquitination of key molecules in the interferon pathway, ‌leading to their degradation‌, thereby inhibiting the antiviral immune response and promoting PCV2 replication. To validate this hypothesis, the regulatory mechanism can be investigated using dual-luciferase assays, co-immunoprecipitation (Co-IP), immunofluorescence, and ubiquitination assays.

Regarding antibacterial immunity, RNF128 expression induces an anergic phenotype in CD4^+^ T cells, enabling *Mycobacterium tuberculosis* to evade surveillance by T cells 40. Moreover, RNF128 activates TBK1 through K63-linked ubiquitination of TBK1 to regulate the secretion of IFN-β, while TBK1 regulates autophagy initiation by phosphorylating Syntaxin17 and promotes autophagy-mediated antibacterial defense 41, 42. Another study reveals that RNF128 specifically binds to IL-3Rα via its PA domain, catalyzes K27-linked polyubiquitination of IL-3Rα, and targets this receptor for lysosomal degradation, thus dampening‌ IL-3/STAT5 signaling 23. Taken together, these studies indicate that RNF128 regulates the progression of infectious inflammation during host antibacterial immune defense.

Regarding antifungal immunity, RNF128-deficient mice exhibit selective expansion of peripheral mature innate lymphoid cells (ILC2s). Accordingly, *Rnf128^-/-^* mice are more susceptible to allergic inflammatory lung diseases upon *Alternaria alternata* (*A. alternata*) infection. This can be attributed to the finding that RNF128 effectively inhibits the number of IL-5- and IL-13-producing ILC2s. More importantly, RNF128 downregulates ST2, the receptor for cytokine interleukin-33 (IL-33), in *A. alternata*-induced allergic lung inflammation 43. Regrettably, this study did not clarify the specific molecular mechanism by which RNF128 inhibits ST2. However, it is speculated that RNF128 may ubiquitinate and degrade ST2 in ILC2s, thereby inhibiting lung inflammation 43.

### 2.5 Regulation of other inflammatory diseases

Acute lung injury (ALI) is a severe disorder characterized by‌ pulmonary inflammation, alveolar-capillary barrier damage, and pulmonary edema. Alveolar macrophages and neutrophils are key mediators in ALI pathogenesis 44, 45. Recent studies reveal that RNF128-deficient mice exhibit enhanced macrophage activation and neutrophil infiltration, which exacerbates lung injury in experimental ALI model 46. Mechanistically, RNF128 mediates K48-linked ubiquitination of Toll-like receptor 4 (TLR4), targeting TLR4 for proteasomal degradation. This event blunts NF-κB phosphorylation and downstream signaling, thus dampening inflammatory response of macrophages. Moreover, RNF128 inhibits neutrophil activation via direct interaction with myeloperoxidase (MPO), lowering MPO expression and enzymatic activity 46. However, it remains unclear whether RNF128 mediates the function of other receptors besides TLR4. Collectively, these findings indicate that RNF128 can antagonize the progression of ALI-related inflammation **(Figure 2 and Table 2)**.

## 3. Regulatory roles and mechanisms of the E3 Ub ligase RNF128 in tumor diseases

The E3 Ub ligase RNF128 regulates tumor progression by mediating substrate protein degradation via ubiquitination. In modulating tumor progression, RNF128 exhibits both pro-tumor and anti-tumor effects **(Table 3)**.

**Figure 4:**
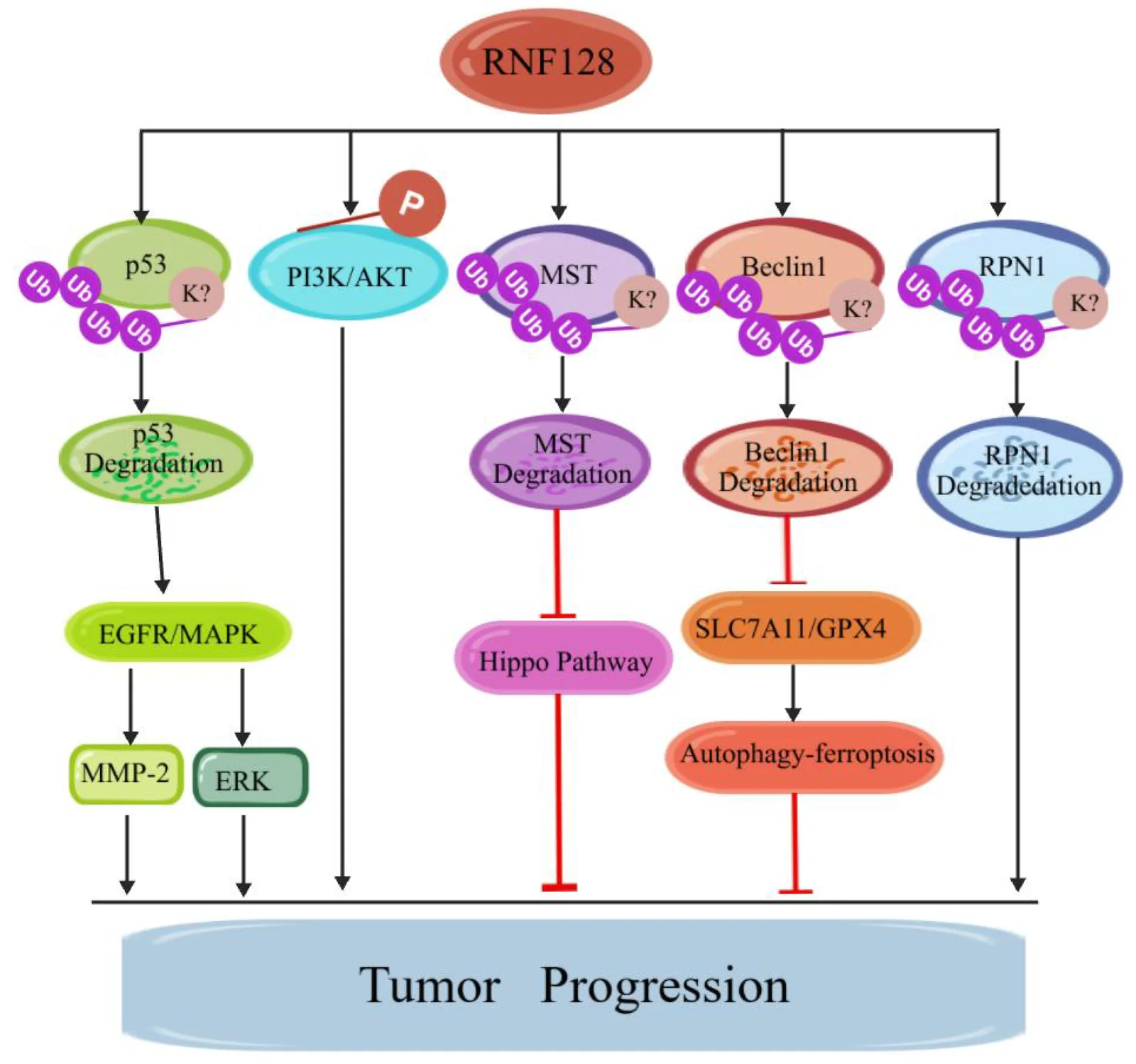
** RNF128 pro-tumor mechanisms.** RNF128 promotes tumor progression by ubiquitinating and degrading p53, MST, Beclin1, and RPN1, thereby regulating the EGFR/MAPK/MMP-2, EGFR/MAPK/ERK, Hippo, and SLC7A11/GPX4 signaling pathways; additionally, RNF128 can activate the PI3K/AKT signaling pathway to further promote tumor progression.

The tumorigenic effects induced by the E3 Ub ligase RNF128 are highly dependent on tumor type. For example, in esophageal squamous cell carcinoma (ESCC), RNF128 binds to p53, enhances epidermal growth factor receptor (EGFR) phosphorylation, prevents the binding between p53 and EGFR, and thereby activates the EGFR/mitogen-activated protein kinase (MAPK or MEK)/matrix metalloproteinase 2 (MMP-2) cascade, thus promoting ESCC invasion and metastasis 47. In HCC tissues, RNF128 expression is much higher relative to that in adjacent normal tissues. Its overexpression promotes proliferation, invasion, and apoptosis resistance of HCC cells both *in vitro* and *in vivo*. Further studies have shown that RNF128 binds to p53 to activate the EGFR/MAPK/extracellular regulated protein kinases (ERK) signaling pathway, thereby facilitating HCC progression 12. Nevertheless, none of these reports have explained how exactly RNF128 interacts with p53. Notably, one study demonstrates that RNF128 targets a subset of p53 for ubiquitination-dependent degradation, whereas the residual p53 is suppressed via an undefined RNF128-driven mechanism 48. This unique regulatory mode deviates from the conventional effect of an E3 Ub ligase on a single substrate, which usually only involves "proteolytic ubiquitination" or "non-proteolytic ubiquitination". Indeed, RNF128 achieves dual-layered suppression toward p53 by triggering both its ubiquitination-dependent degradation and functional inactivation. This dual regulation may involve RNF128-induced conformational changes in p53 or interference with coactivator binding. However, the dual regulatory mechanism remains elusive, representing a critical research gap that warrants urgent investigation. In CRC, RNF128 expression is significantly upregulated at both mRNA and protein levels. Knockout of RNF128 markedly attenuates the malignant phenotype of CRC cells *in vitro* 49, 50. Furthermore, studies have demonstrated that RNF128 promotes malignant biological behavior of CRC cells by regulating the phosphoinositide 3-kinase (PI3K)/protein kinase B (PKB, also known as AKT) signaling pathway 49. Nevertheless, a limitation of this study lies in its failure to elucidate the specific mechanisms by which RNF128 activates the PI3K/AKT pathway. In another study, RNF128 has been shown to mediate ubiquitination-dependent degradation of mammalian STE20-like kinase (MST) and repress Hippo signaling, thereby promoting proliferation, migration, and invasion of CRC cells 51. Similarly, it has been reported that RNF128-mediated ubiquitination and degradation of ribophorin I (RPN1) promotes CRC progression 52. In gastric cancer (GC) cells, RNF128 is highly expressed. Knockout of RNF128 significantly inhibits GC cell proliferation and increases intracellular autophagic flux and lipid peroxide levels 53. Subsequent studies have demonstrated that RNF128 mediates the ubiquitination and degradation of Beclin1, suppresses the Beclin1/solute carrier family 7 member 11 (SLC7A11)/glutathione peroxidase 4 (GPX4) axis, reduces ferroptosis, and thus promotes GC progression 53. This study is the first to link RNF128 to the autophagy-ferroptosis crosstalk pathway, but lacks validation at the clinical tissue level and research on its impact on patient survival **(Figure 4)**.

**Figure 5:**
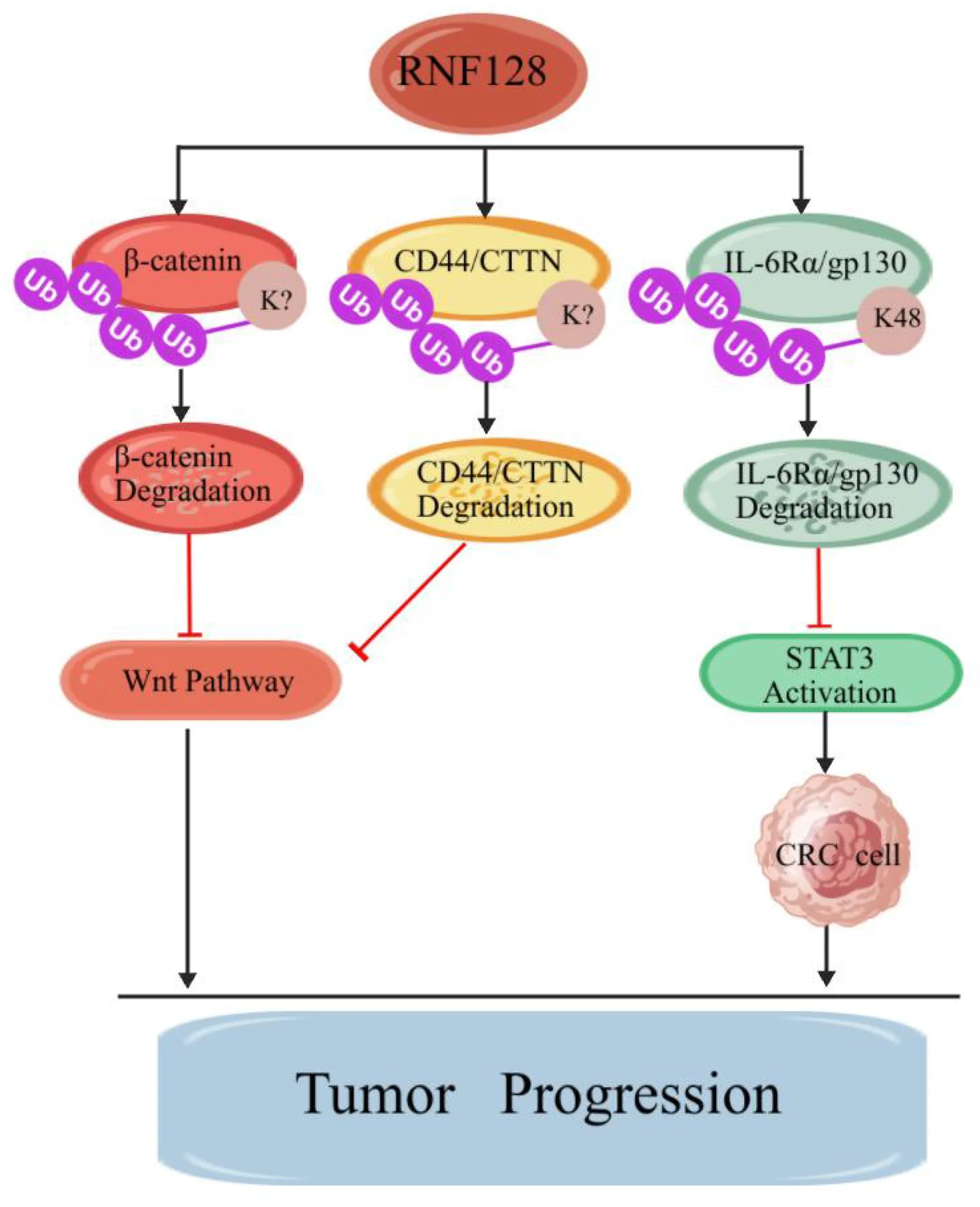
** RNF128 anti-tumor mechanisms.** RNF128 regulates signaling pathways such as Wnt and STAT3 by ubiquitinating and degrading β-catenin, CD44/CTTN, and IL-6Rα/gp130, thereby inhibiting tumor progression.

In contrast to its predominant oncogenic role in gastrointestinal malignancies, RNF128 overexpression inhibits CRC cell proliferation, migration, and invasion, while RNF128 knockout promotes the malignant phenotypes of CRC cells. Subsequent studies have confirmed that RNF128 regulates β-catenin ubiquitination, inhibits canonical Wnt transcriptional activity, and suppresses the Wnt signaling pathway to exert context-dependent tumor-suppressive effects 12. A similar mechanism has been observed in RNF128-mediated inhibition of prostate cancer progression 54. Another study has demonstrated that RNF128 can also inhibit the Wnt signaling pathway by mediating the ubiquitination and degradation of CD44 and cortactin (CTTN), thus suppressing malignant melanoma progression 55. Beyond suppressing the Wnt signaling cascade, RNF128 can also degrade IL-6Rα and its co-receptor membrane glycoprotein gp130 via the Ub-lysosome pathway, and block the IL-6-STAT3 signaling pathway to suppress CRC progression 21 **(Figure 5)**.

RNF128 exhibits a diametrically opposite functional role in CRC, and this seemingly contradictory phenomenon cannot be simply attributed to experimental errors, reflecting the environment-dependent regulatory characteristics of E3 Ub ligase function. An analysis of existing research evidence reveals that the confounding effects of multiple key regulatory factors, combined with variations in experimental systems, collectively mediate the functional heterogeneity of RNF128 in CRC. The variation in cell line selection is the primary reason. Studies investigating pro-tumor effects predominantly utilized cell lines with high RNF128 expression levels, such as SW480, SW1116, and HCT116, for functional analyses. These cell lines exhibit relatively low activity in signaling pathways, including the Hippo pathway. Conversely, studies investigating anti-tumor effects predominantly utilize CRC cells with low RNF128 expression, which exhibit high activity in signaling pathways such as the Wnt and inflammatory signaling pathways. In overexpression experiments, RNF128 is expressed at levels exceeding physiological thresholds, potentially leading to the ubiquitination and degradation of a large number of low-affinity substrates, thereby inducing aberrant functions that are absent under physiological conditions. In addition, there are significant differences between plasmid-overexpressed proteins and endogenous proteins in terms of subcellular localization and post-translational modification status. Changes in the protein levels of key substrates were not found to correlate with phenotypic changes. Furthermore, existing studies generally suffer from limited clinical sample sizes, and stratified analyses based on tumor stage and inflammatory background have not been performed. Finally, differences in experimental methodology further amplify this divergence.

## 4. Mechanistic hypotheses for the E3 Ub ligase RNF128

Given the contradictions and methodological limitations identified in the existing evidence, this paper proposes three testable hypotheses to reconcile the aforementioned controversies. Firstly, we propose the tumor stage-dependent functional switching hypothesis. In the early stages of CRC, RNF128 primarily suppresses STAT3-mediated inflammatory signaling by degrading the IL-6Rα/gp130 complex, while simultaneously inhibiting aberrant proliferation signaling through the degradation of β-catenin, thereby exerting its tumor-suppressor gene function at this stage. When tumors progress to an advanced stage, the Hippo pathway becomes partially inactivated due to promoter methylation or mutations. This leads to a significant decrease in MST protein levels and an increase in the molar ratio of RNF128 to residual MST proteins. Under such conditions, further suppression of the Hippo pathway occurs primarily via degradation of MST proteins, thereby promoting tumorigenesis. This hypothesis can be tested by conditionally knocking out RNF128 at distinct time points in an AOM/DSS-induced CRC model. Secondly, we propose the molecular subtype-dependent substrate selection hypothesis. In inflammation-enriched CRC, the IL-6-STAT3 pathway is hyperactivated, with protein levels of IL-6Rα and gp130 markedly exceeding those of MST. Under these conditions, RNF128 preferentially binds to and degrades inflammatory receptors, thereby exerting a tumor-suppressive effect. In CRC with the inactivated Hippo signaling pathway, RNF128 abundance is significantly higher than that of MST. The degradation of MST by RNF128 exhibits a pro-tumor effect. This hypothesis can be validated by systematically comparing the interaction proteome of RNF128 in different molecular subtypes of CRC cell lines within the same isogenic background. Finally, we propose the dose-dependent substrate competition hypothesis. The substrate selection of RNF128 follows the principle of competition between abundance and affinity. At the physiological level, high-affinity substrates (IL-6Rα, gp130, and CD44) are preferentially ubiquitinated, during which RNF128 primarily exerts anti-inflammatory and anti-tumor functions. When gene amplification or transcriptional upregulation causes RNF128 expression to exceed a certain threshold, high-affinity substrates become saturated, while low-affinity substrates (MST and RPN1) begin to undergo extensive ubiquitination. Under these conditions, pro-tumor effects may arise. This hypothesis can be validated using gradient experiments that precisely control the expression dosage of RNF128 in conjunction with quantitative proteomics. The validation of these hypotheses will not only resolve the functional controversies surrounding RNF128 in CRC but will also provide a methodological paradigm for studying the bidirectional functions of other E3 Ub ligases.

## 5. Conclusion

The E3 Ub ligase RNF128 plays an important role in regulating the development of inflammatory diseases and tumors by mediating protein ubiquitination through its specific structural domains. It exerts both pro-inflammatory and anti-inflammatory effects in inflammatory diseases, as well as pro-tumorigenic and anti-tumorigenic effects in neoplastic diseases. These seemingly contradictory functional modes are not closely associated with specific cell types. They primarily depend on the particular target receptors or substrate proteins ‌recognized by RNF128, as well as their specific ubiquitination linkage patterns. More importantly, analysis of RNF128 mechanisms in inflammation and tumors reveals that, in addition to mediating protein ubiquitination and degradation, RNF128 also functions in non-proteolytic ubiquitination **(Table 2 and Table 3)**. Moreover, RNF128 expression levels are closely correlated with disease severity and clinical prognosis, highlighting its potential as a promising marker for the diagnosis, prognostic evaluation, and therapeutic intervention of inflammatory diseases and tumors.

## 6. Key issues and future directions

Although significant breakthroughs have been achieved in current research on RNF128 regarding inflammatory diseases and tumors, numerous scientific challenges persist. Specifically, the spatial conformational changes of RNF128 remain structurally undefined; its underlying mechanisms lack comprehensive elucidation; and the pathway for its clinical translation remains unclear. Therefore, future research directions include: 1) Investigating the structure-function relationship of RNF128 to analyze the impact of its conformational changes on substrate protein selectivity; 2) Employing multi-omics integration analysis to establish a three-dimensional regulatory network of RNF128, disease, and the microenvironment, thereby improving the functional profile of this molecule; 3) Exploring therapeutic strategies targeting RNF128 to facilitate the translation of basic research into clinical practice; 4) Strengthening interdisciplinary collaborative research to promote the innovative development of RNF128-related studies.

Leveraging an E3 Ub ligase-focused research and development platform, targeted modulation of RNF128 using small-molecule inhibitors and proteolysis-targeting chimeras (PROTACs) is underpinned by robust theoretical and technical foundations. This strategy may offer innovative avenues for precision intervention against inflammatory disorders and tumors. However, this targeted therapy system still faces numerous challenges that require urgent resolution, including off-target effects induced by the high homology of E3 Ub ligases and the low efficiency of tissue-specific drug delivery, both of which markedly undermine the safety and therapeutic efficacy. Notably, RNF128 mediates critical physiological processes in normal tissues, including T cell immune tolerance and tissue homeostasis. Consequently, targeted therapy against RNF128 may disrupt its physiological immunoregulatory role and induce off-target toxicity in healthy tissues. Abnormal RNF128 expression is associated with tumor progression, suggesting its notable potential as a prognostic biomarker; however, evidence supporting its value as a predictive biomarker remains limited. Furthermore, marked inter-tumor heterogeneity and the lack of large-scale clinical cohorts hinder its translational utility. Addressing these risk-related challenges requires combined clinical and experimental validation to assess the performance of this RNF128-derived biomarker and balance therapeutic benefits against the risk of normal-tissue toxicity. At present, clinical trials of other E3 Ub ligases have accumulated sufficient data and insights, confirming that a single broad-spectrum targeted drug may readily disrupt normal signaling and induce *in vivo* toxic side effects. Precise targeting and personalized functional regulation are core prerequisites for E3 Ub ligase targeted therapy. RNF128 exerts anti-inflammatory and anti-tumor effects in chronic inflammation and some tumors, and can be moderately activated to curb disease progression. In models of various malignant tumors and acute inflammation, RNF128 mediates the activation of pro-tumor and pro-inflammatory signaling pathways, requiring targeted inhibition via PROTACs or specific small-molecule inhibitors. This bidirectional regulation strategy serves as the prerequisite for breaking through the bottleneck of RNF128 clinical translation and achieving precise treatment. In summary, the E3 Ub ligase RNF128 plays a critical role in the progression of inflammatory diseases and tumors, endowing it with profound basic research value and promising clinical application prospects.

## Figures and Tables

**Table 1:** Common ubiquitination-dependent functions of RNF128 and its homologous proteins

E3 ligases	Substrates	Ub-chain types	Functions	Ref.
RNF128	IL-6Rα, gp130	K48-linked	RNF128 mediates the degradation of IL-6Rα and gp130 via the lysosomal pathway.	21
	TBK1	K63-linked	RNF128 promotes K63-linked ubiquitination of TBK1, leading to TBK1 phosphorylation and activation.	22
	IL-3Rα	K27-linked	RNF128 mediates the degradation of IL-3Rα via the lysosomal pathway.	23
RNF167	RIG-I, MDA5	K6-linked, K11-linked	RNF167 catalyzes the atypical K6- and K11-linked polyubiquitination of RIG-I/MDA5, and subsequently directs them for degradation through dual proteolytic pathways of both lysosomal and proteasomal.	24
RNF149	CD63	K29-linked	RNF149 mediates the K29 ubiquitination-dependent degradation of CD63.	25

**Table 2:** The roles of RNF128 in inflammatory disorders

Diseases	RNF128 expression	Mechanisms	Cells	Ubiquitination linkage types	Substrate proteins	Ubiquitination degradation	Functions	Ref.
Allergic asthma	low	RNF128 inhibits IL-2R desensitization by blocking the activity of cullin5 ligase, thereby stabilizing the function of Tregs.	Tregs	Not reported	Cullin5-K724	Yes	Inhibition	26
SLE	low	RNF128 may enhance the suppressive function of Tregs.	Tregs	Not reported	Not reported	Not reported	Inhibition	27
EAE	low	NK cells expressing RNF128 induce T cells anergy.	NK cells	Not reported	Not reported	Not reported	Inhibition	29
Colitis	low	RNF128 alleviates colitis by inhibiting IL-6-STAT3 signal transduction.	Macroph-ages	K48	IL-6R, gp130	Yes	Inhibition	21
	low	RNF128 exerts intestinal protective effects by regulating IL-10R to support regulatory immune responses and shaping the microbiota.	CD4^+^ T cells	Not reported	IL-10R	Not reported	Inhibition	32
	low	RNF128 alleviates colonic inflammation by promoting the autophagic degradation of K63-linked ubiquitination of S100A8.	Macroph-ages	K63	S100A8	Autophagic degradation	Inhibition	33
AS	High	RNF128 promotes K63-linked polyubiquitination on SRB1 in macrophages, thereby exacerbating AS.	Macroph-ages	K63	SRB, oxLDL	Non-proteolyt-ic ubiquitination	Promotion	37
Virus infection	High	RNF128 promotes the expression and secretion of IFN-β through K63-linked ubiquitination of TBK1, thereby exerting antiviral immunity.	Macroph-ages and THP-1 cells	K63	TBK1	Non-proteolyt-ic ubiquitination	Inhibition	22
IAV infection	High	RNF128 controls the replication and infection of IAV by enhancing the degradation of viral NP.	Epithelia-l cells of the lung	Not reported	NP	Yes	Inhibition	38
PCV2	High	RNF128 inhibits the production of IFN-β and enhances the viral replication of PCV2 in PK15 cells.	PK15 cells	Not reported	Not reported	Not reported	Promotion	39
*Mycobacter-ium tuberculosis* infection	High	RNF128 induces unresponsiveness in CD4^+^ T cells.	CD4^+^ T cells	Not reported	Not reported	Not reported	Inhibition	40
Adherent- invasive Escherichia coli (AIEC) infection	low	RNF128 regulates the secretion of IFN-β by activating TBK1 through ubiquitination modification, and the activated TBK1 in turn phosphorylates Syntaxin17 to initiate autophagy, thereby mediating antibacterial defense.	Macroph-ages	Not reported	TBK1	Non-proteolyt-ic ubiquitination	Inhibition	41
Sepsis	High	RNF128 negatively regulates the IL-3/STAT5 signaling pathway by facilitating K27-linked polyubiquitination of IL-3Rα.	Macroph-ages	K27	IL-3Rα	Yes	Inhibition	23
*A. alternata* infection	High	RNF128 may degrade ST2 through ubiquitination, thereby inhibiting pulmonary inflammation.	ILC2s	Not reported	ST2	Not reported	Inhibition	43
ALI	High	RNF128 reduces macrophage inflammation by regulating the activation of the TLR4-NF-κB signaling pathway.	Macroph-ages	K48	TLR4	Yes	Inhibition	44

**Table 3:** The roles of RNF128 in tumors

Diseases	RNF128 expression	Mechanisms	Cells	Ubiquitination linkage types	Substrate proteins	Ubiquitination degradation	Functions	Ref.
ESCC	High	RNF128 drives the invasion and metastasis of ESCC cells through the EGFR/MAPK/MMP-2 signaling pathway.	ESCC Cells	Not reported	p53	No	Promotion	47
HCC	High	RNF128 promotes the progression of HCC by activating the EGFR/MEK/ERK signaling pathway.	HCC cells	Not reported	p53	No	Promotion	11
CRC	High	RNF128 promotes the progression of CRC by regulating the PI3K/AKT pathway.	CRC cells	Not reported	Not reported	Not reported	Promotion	49
	High	RNF128 promotes the progression of CRC by inhibiting the Hippo signaling pathway.	CRC cells	Not reported	MST	Yes	Promotion	51
	High	RNF128 promotes the migration of CRC cells by downregulating RPN1.	CRC cells	Not reported	RPN1	Yes	Promotion	52
	low	RNF128 inhibits the malignancy of CRC cells by suppressing the Wnt/β-catenin signaling pathway.	CRC cells	Not reported	β-catenin	Ubiquitination but unspecified degradation	Inhibition	12
	low	RNF128 attenuates the progression of CRC by inhibiting IL-6-STAT3 signal transduction.	Macrophages	K48	IL-6Rα/gp130	Yes	Inhibition	21
GC	High	RNF128 promotes the malignant progression of GC by inhibiting the Beclin1/SLC7A11/GPX4 axis.	GC cells	Not reported	Beclin1	Yes	Promotion	53
Prostate cancer	Low	RNF128 inhibits the progression of prostate cancer by suppressing the Wnt/β-catenin signaling pathway.	Prostate cancer cells	Not reported	β-catenin	Not reported	Inhibition	54
Melanoma	low	RNF128 inhibits Wnt signal transduction by ubiquitinating and degrading CD44/CTTN, thereby suppressing melanoma progression.	Melanoma cells	Not reported	CD44/CTTN	Yes	Inhibition	55
